# The impact of imposter phenomenon: an unmet need in the education of healthcare personnel- a cross-sectional study

**DOI:** 10.1186/s12909-025-08349-3

**Published:** 2025-11-21

**Authors:** Ida Marie Heggem, Gabriela Wale Soto, Hans Jørgen Guthe, Omar Hikmat

**Affiliations:** 1 Department of Paediatric and adolescent medicine, Haukeland University Hospital, Bergen, Norway; 2 Department of Education, University of Bergen, Bergen, Norway; 3 Western Norway University of Applied Science, Bergen, Norway; 4 Department of Clinical Science (K2), University of Bergen, Bergen, Norway

**Keywords:** Burnout, Education, Imposter, Nurses, Physicians

## Abstract

**Background:**

Imposter Phenomenon (IP) is a phenomenon characterised by self-doubt and fear of exposure by peers as a fraud. We aimed to assess the frequency and the severity of IP in medical personnel.

**Methods:**

A cross-sectional, descriptive study, the participants were recruited from Haukeland University Hospital, and Western Norway University of Applied Science. The Clance Imposter syndrome scale (CIPS) was implemented to verify the presence and severity of IP.

**Results:**

A total of 116 health personnel were recruited, 62 physicians and 54 nurses. IP was identified in 89% of participants, 60% with significant to intense degree. IP was significantly more prevalent in females as compared to males and was identified in 82% and 90% of physicians and nurses respectively. IP severity decreased with increasing experience.

**Conclusion:**

The majority of our study population had some degree of IP, highlighting the need to address IP both in pre-and post-graduate education of health personnel.

## Background

 Imposter Phenomenon (IP) was first described by Clance et al. in 1978 among high-achieving women [1], specifically women with higher education and in positions of power or influence in academia. It is now known to occur in all genders, professional fields and levels of experience [2–4]. IP is not recognised as a psychological disorder and is not listed in The International Classification of Diseases (ICD) or in Diagnostic and Statistical Manual of Mental Disorders, 4th Edition (DSM IV), but is considered rather as a psychological phenomenon. IP is characterised by feeling incompetent and undeserving, doubting one’s achievements, leading to a fear of being discovered as a fraud [1]. Achievements are often attributed to external factors such as luck and good timing, not as a result of hard work and skill and often despite evidence of the opposite. The phenomenon is particularly prevalent in the medical professions and amongst students of health sciences [5, 6], which is at least partially exacerbated by the nature of the work and the structure of the education. While the hierarchy and the art of medicine requires humility and insight into one’s limitations, high levels of IP can lead to anxiety, depression and burnout [5, 7]. In a profession that already has increased risk of mental illness and suicide compared to the general population [5, 7, 8], addressing the phenomenon and attempting to reduce the long-term effects on the individual’s mental wellbeing is increasingly important. Today`s working environment with an *i*ncreasing patient load, lack of personnel and incomplete software systems, as well as the desire to have a semblance of work-life-balance, puts health care personnel under consistently high pressure. Increased knowledge about the IP, its effects and how to overcome it may alleviate some of the pressure and reduce morbidity.

Several studies and case series on medical students and medical professionals have been published addressing the phenomenon since it was first described, primarily in the United States and the United Kingdom [5, 9, 10]. One study in Denmark looked at confidence in the operating room amongst residents in obstetrics and gynaecology, using CIPS as one of the elements of their questionnaires [11]. Although there are many studies focusing on mental health among health care workers it seems to be few descriptive studies focusing on the IP itself. In this study, we aimed to assess the frequency and severity of IP in a population of medical personnel from western Norway. Furthermore, we aimed to investigate if there are any differences in the frequency and severity of IP between physicians working in the clinical field versus those within the laboratory field, and between physicians and nurses with different level of experience.

## Methods

### Study design and population

In this cross-sectional descriptive study, physicians (consultants and residents) were recruited from both a clinical and a laboratory department at Haukeland University Hospital. Nurses were recruited from a clinical department, while nurses in subspecialisation were recruited from Western Norway University of Applied Science.

### Study data

Study data were collected via the Clance Imposter syndrome scale (CIPS), a standardised self-report questionnaire that has been validated and is used in several studies [12]. It consists of 20 statements in a 5-point Likert format, from 1 = not at all true, to 5 = very true. It results in a score between 10 and 100, which is directly proportional to the level of manifestation of IP [1, 12]. The lower the score, the less severe levels of IP. The questionnaire was distributed electronically via email or through a QR code that participants could access using their personal devices. The survey collected demographic and professional information, including participants’ profession, age, gender, and years of clinical experience. The questionnaire link was distributed to all participants within the participating departments, encompassing individuals in both permanent and temporary positions. The survey was conducted between September 2023 and November 2023. Data sampling was performed using a stratified random approach according to profession, including physicians in clinical departments, physicians in laboratory fields, and nurses. This method ensured balanced representation across professional groups and enhanced the interpretability of comparisons between them. Study data were collected using Microsoft Forms (MF), generated and stored securely at University of Bergen.

### Statistical analysis

Descriptive data analysis was performed using statistical software package SPSS version 23.0. The severity of IP in each individual was based on their CIPS score (Table 1). CIPS score was compared between groups (gender, length of experience, residents, consultants, clinical and laboratory fields, and nurses) using independent *t*-test. P-values of < 0.05 were considered significant.

**Table 1:** The CIPS score showing corresponding IP severity level

CIPS Score	Severity level of Imposter Phenomenon
0–40	None to mild
41–60	Moderate
61–80	Significant
81–100	Intense

## Results

### Study population

**Fig. 1:**
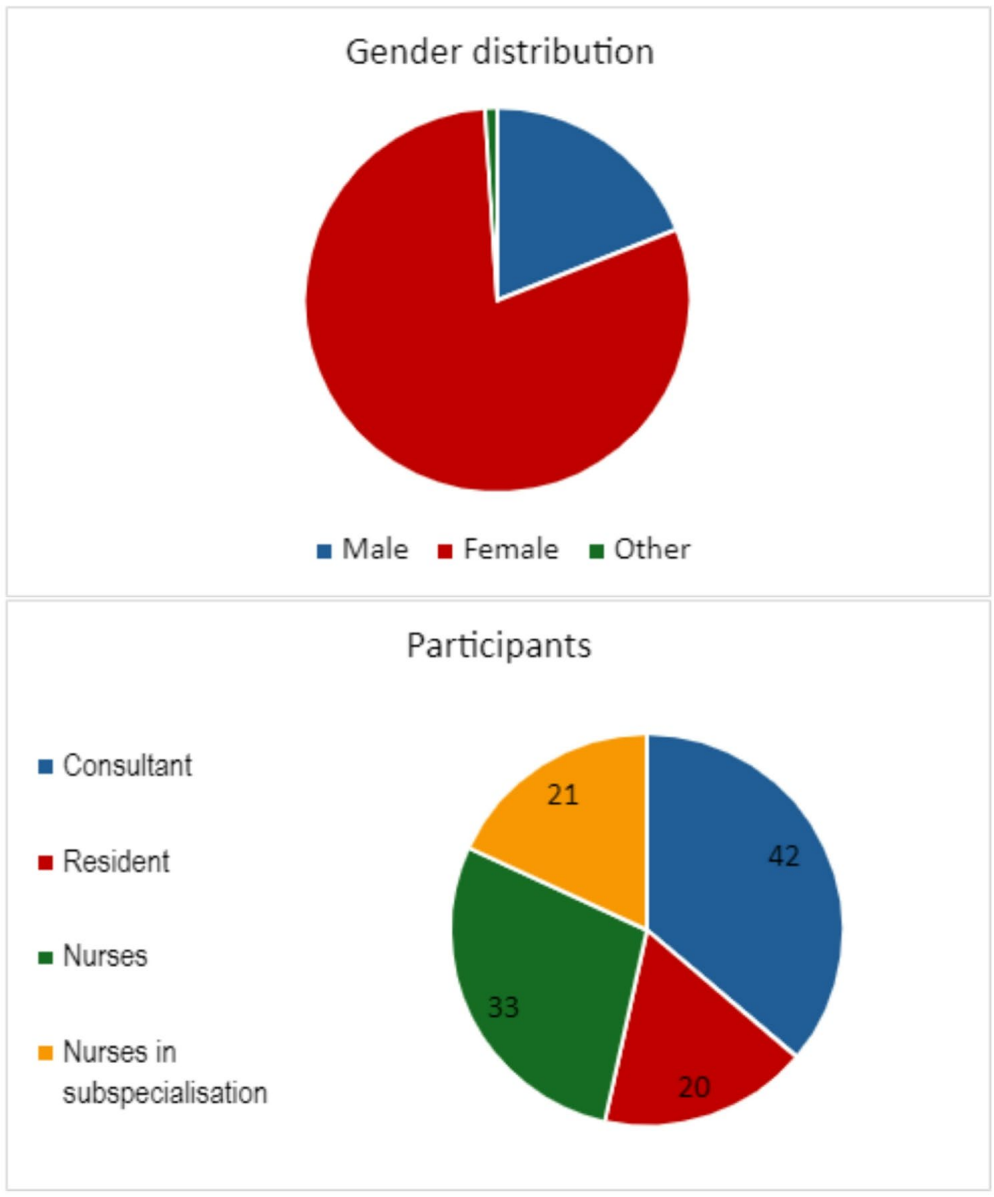
**A**. Gender distribution in the study population. **B.** Profession and level of experience in the study population

The questionnaire yielded responses from 116 individuals. The female to male ratio was 4:1, reflecting the current composition of gender among health care personnel in Norway. One participant did not specify their sex in the questionnaire. Overall, 20 residents and 42 consultants responded, nearly equally divided between the clinical field and the laboratory field with 32 and 30 participants respectively. A total of 54 nurses were recruited, 33 were specialist nurses and 21 were in the process of specialisation (Fig.1).

### Prevalence and severity of IP overall

**Fig. 2:**
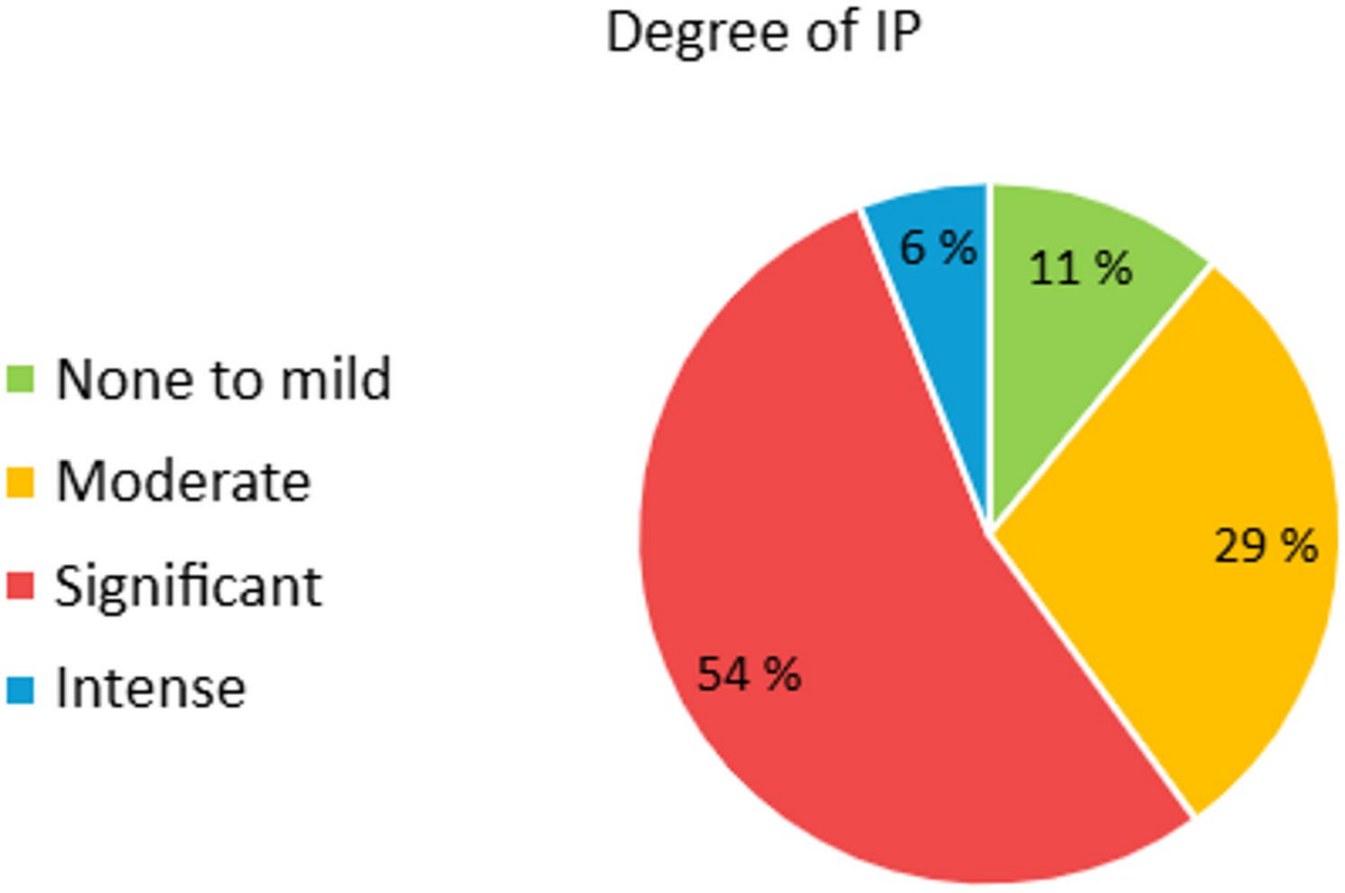
Severity of IP in the study population

In total, 89% (no. = 103) had moderate to intense degree of IP, with 54% (no. = 62) and 6% (no. = 7) demonstrating significant and intense degree of IP respectively. In our study population females were significantly (*p* = 0.005, 95% CI: −16,224 to-2,896) more severely affected compared to males. Mean CIPS score was 63 (SD = 14) (significant IP) for females, and 53 (SD = 14) for males (moderate IP). Summary of the results is provided in Table 2 (Fig.2).

**Table 2:** Summary of the findings stratified by gender, professional role (physicians, residents, nurses, and nurses in subspecialisation), and years of experience. CIPS: Clance imposter syndrome scale. IP: imposter phenomena

Category	No.	Mean CIPS score	IP grade
Male	22	53(SD 14)	Moderate
Female	93	63(SD 14)	Significant
Consultants ≤ 10 years of experience	9	60(SD 9)	Moderate
Consultants > 10 years of experience	33	58(SD 15)	Moderate
Physicians in training (residents) ≤ 2 years of experience	5	62(SD 19)	Significant
Physicians in training (residents) > 2 years of experience	15	68(SD 17)	Significant
Physicians in training (residents) regardless of experience	20	66(SD 17)	Significant
Consultants regardless of experience	42	58(SD 14)	Moderate
Physicians laboratory field	30	62(SD 15)	Significant
Physicians clinical field	32	60(SD 16)	Significant
Nurses in subspecialisation ≤ 5 years of experience	13	68(SD 11)	Significant
Nurses in subspecialisation > 5 years of experience	8	53(SD 12)	Moderate
Nurses < = 10 years of experience	15	62(SD 14)	Significant
Nurses > 10 years of experience	18	58(SD 15)	Moderate

### IP in physicians versus nurses

**Fig. 3:**
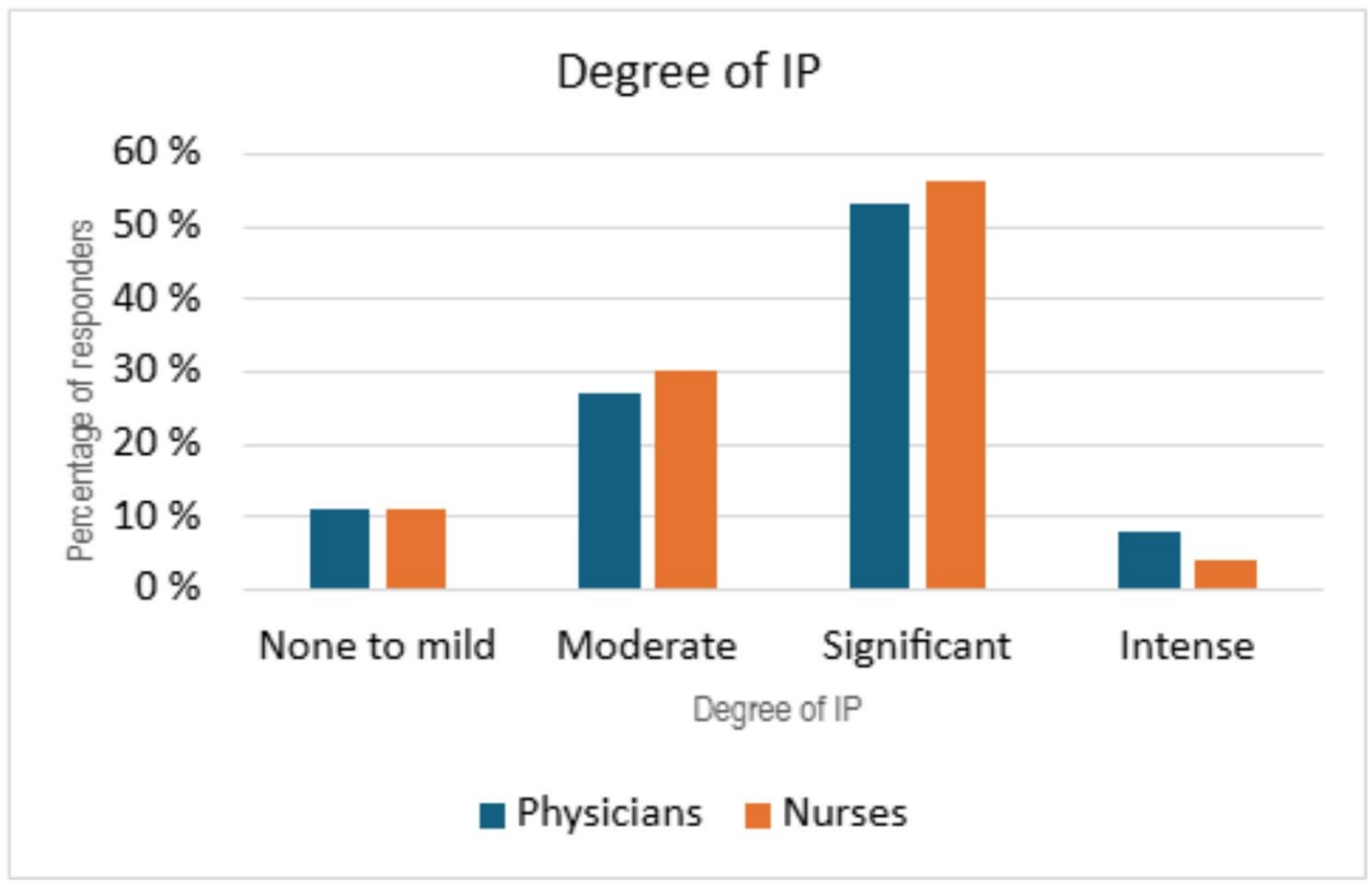
IP severity in physicians versus nurses

The prevalence of IP was similar among both nurses and physicians. Physicians (both consultants and residents) had slightly higher CIPS score than nurses with 53% (no. = 33) and 8% (no. = 5) reporting significant and intense level of IP respectively. Amongst nurses the score was 56% (no. = 30) and 4% (no. = 2) for significant and intense level of IP respectively, however a higher number reported moderate levels of IP. Even so, in both groups 89% had moderate or higher degree of IP (Fig.3).

### IP in physicians working within the clinical field versus laboratory field

**Fig. 4:**
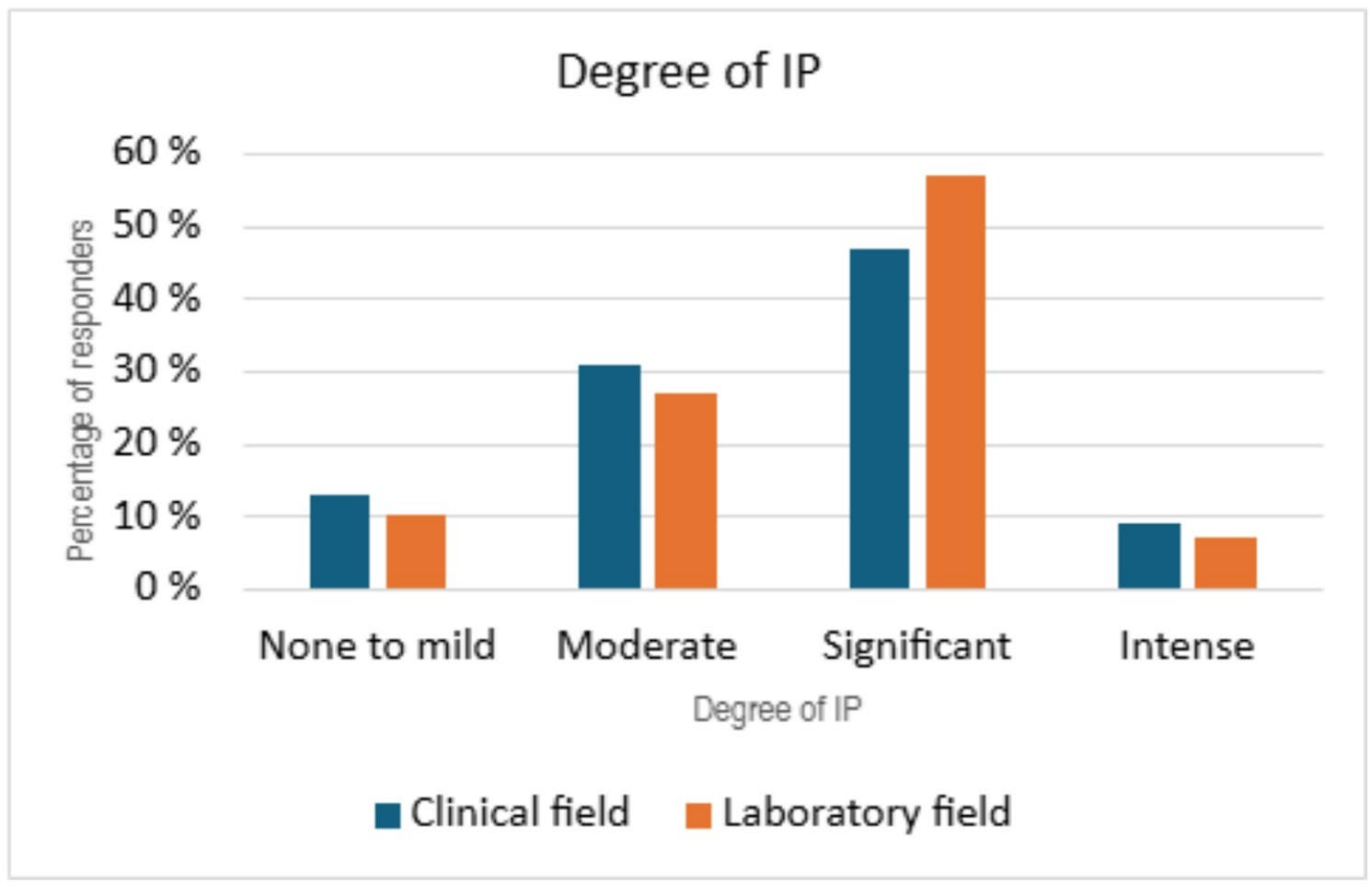
IP severity in a clinical field and a laboratory field

IP was more prevalent and severe in physicians working in a laboratory field, rather than a clinical one. In the laboratory field, 64% (no. = 19) had intense or clinically significant levels of IP, whereas in the clinical field it was 56% (no. = 18). There was, however, no statistically significant difference between these two groups (*p* = 0.57, 95% CI:−9,99 to 5,57) (Fig. 4).

### IP in consultants versus residents

**Fig. 5:**
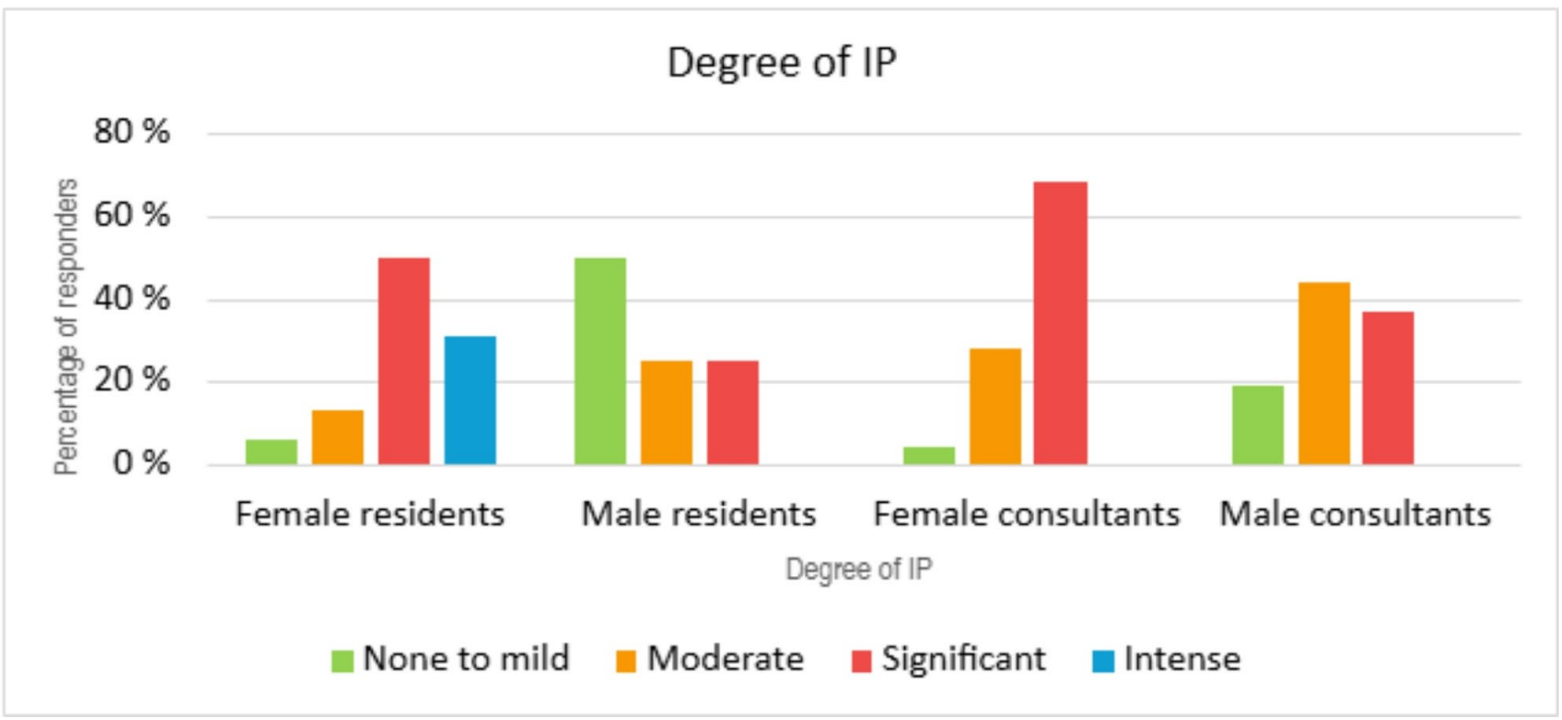
IP severity in residents and consultants, adjusted for gender

CIPS score was higher for residents than consultants, where no consultants reported intense levels of IP, as opposed to 25% (no. = 5) of residents (Fig. 5). Furthermore, the mean CIPS score was 58 (SD = 14) for consultants, compared to 66 (SD = 17) for residents, however the difference was not statistically significant (*p* = 0.055, 95% CI: −15,99 to 0,183). Both female consultants and residents had higher levels of IP compared to their male colleagues (Fig. 5*)*. Close to one in three female residents scored the highest level of IP equal to an intense degree, as opposed to none of the male residents. 50% (no = 2) of male residents scored levels of none to mild IP. Almost 70% (no = 17) of female consultants had significant levels of IP, whereas 37% of male consultants reported the same. Overall, the severity of IP abated with increasing levels of experience.

### IP in nurses versus nurses in subspecialisation

**Fig. 6:**
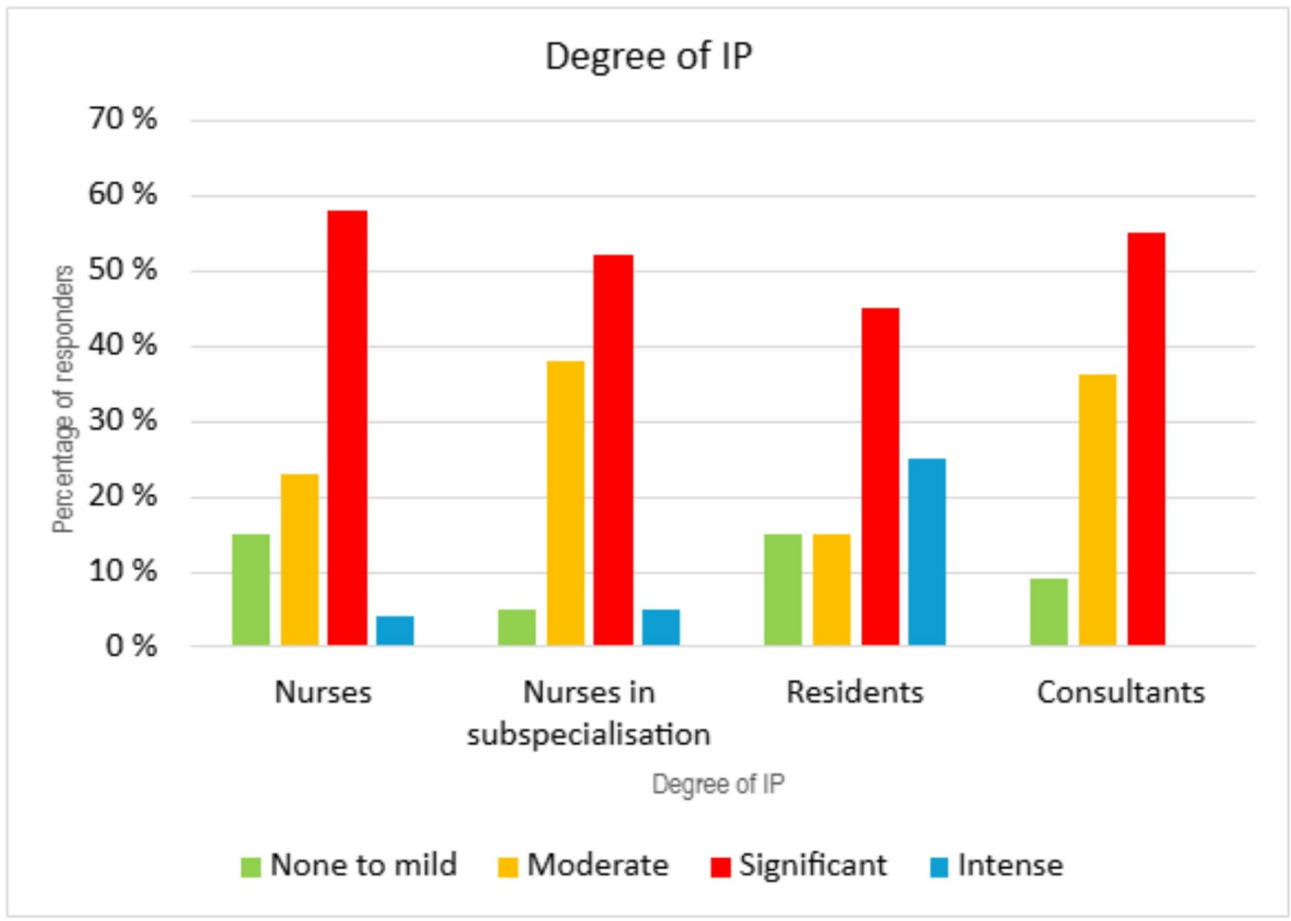
CIPS score and IP severity for all professions

The mean CIPS score for nurses was 60 (SD = 13), while it was 63 (SD = 11) for nurses in subspecialisation (Fig. 6). The difference between these two groups was significant (*p* = 0.009, 95% CI: 4,27 to 25,895). For both groups the CIPS score decreased with increasing experience, and the trend was most pronounced for nurses in subspecialisation. The prevalence of moderate, significant or intense IP were higher for nurses in subspecialisation.

## Discussion

Our study shows that IP is highly prevalent in our study group of nurses, nurses in subspecialisation, residents and consultants. This is concordant with other studies focusing on health care personnel [2, 3]. 

Health care personnel have a unique responsibility, where choices might have greater repercussions than in many other fields of work. The complexity of the work often requires a swift verdict that have a direct impact on patient’s lives. Physicians and nurses experiencing IP might question their diagnosis, treatment plans, or clinical judgement, even when these are based on solid medical knowledge, guidelines and experience. The pressure can exacerbate IP, leading to self-doubt, anxiety, emotional burnout, depression and even decision aversion [2, 3, 5–7, 13]. 

As many as 89% of the participants have some level of IP, illustrating that IP is prevalent in health personnel. However, one can argue that some level of uncertainty and reflection is important as health care personnel. One should continually evaluate if one has indeed reached the right diagnosis, if the treatment is yielding the desired results, or if you should seek expert opinion. Moreover, on an individual level one should continually seek knowledge, improvement and growth, as well as encourage an open discussion and a cooperative working environment. It can thus be discussed if the scale should be slightly modified, as the lowest possible score is not 0 but 10, and if the cut-off for moderate and severe levels of IP should be higher. Further studies are needed to clarify which CIPS score and severity levels of IP are directly correlated with more severe mental health issues such as burnout, anxiety and depression.

In our study population over 30% of female residents have intense levels of IP, and 70% of female consultants have significant levels of IP, which is in concordance with the literature [2, 3, 5–7, 14]. Some of this may be due to prevailing traditional gender views in society, but as the number of female health care professionals increases and the gender balance has shifted, some studies suggests that it is rather the perfectionist personality types of those individuals that go into this line of work that are more predisposed to IP [2, 6].

Even though the severity of IP decreases with increasing experience, it is illustrative to find that it is still significant amongst consultants. It warrants reflection upon the nature of medical education, as well as illustrating the need for increased candour among colleagues. Medical school encourages academic competitiveness, sometimes publicly displaying examination results or rewarding excellence with prices, scholarships, first pick of internships or research opportunities. Not surprisingly, several studies show that the levels of IP are higher at the beginning of medical training and when one is about to be responsible for one’s own patients [5, 6, 15]. Among physicians, nurses and other professions, the levels are naturally higher when one has started a new job or a new project [2, 3]. In health care, as medicine becomes increasingly specialised, newly certified personnel is required to gain experience from a multitude of different fields and workplaces and will constantly be new and inexperienced for a period of time. Addressing the phenomenon in the medical education and at the beginning of the career, as well as consistently working on it with a mentor and/or a group of peers seems warranted with the results our study shows. Perhaps it should also be part and parcel of becoming a mentor or supervisor for new colleagues.

An interesting finding of this study was that laboratory-based physicians exhibited higher levels of impostor phenomenon compared to their clinically oriented counterparts. Several factors may contribute to this difference. Laboratory-based physicians often work in environments with limited direct patient interaction and receive less immediate or personalized feedback on their professional performance, which may reduce opportunities for external validation. Furthermore, the nature of laboratory medicine places strong emphasis on diagnostic precision and accuracy, where even minor errors can have significant consequences, potentially fostering heightened self-scrutiny and doubt. The relative professional isolation and the indirect nature of their clinical contributions may further intensify feelings of inadequacy or undervaluation, thereby amplifying impostor tendencies within this group.

Since our study is descriptive and cross sectional, further studies are warranted that look at overcoming IP and the presumably dynamic nature of IP through a career, such as longitudinal studies. Moreover, studies specifically regarding the different modalities and effects of treatment for IP is needed. Several studies explore cognitive behavioural therapy [1, 15, 16] through individual or group therapy, whereas another study looked at “empathy rounds” as an arena where experiences could be shared and discussed between residents and consultants [17]. Lay literature discusses the importance of feedback; both accolades, encouragement and constructive criticism is important to combat IP [1, 15, 16]. Studies also demonstrate the importance of a debrief after particularly challenging experiences, as well as the importance of being earnest about one’s errors [18]. Even so, neither long term studies nor randomised clinical trials regarding treatment of IP have yet been published.

One of the limitations of the study is that it was sent out as an email to a distribution list, thus the number of people who received the email is unknown, likewise the number of people who have seen the QR-code. Hence, we do not know the response rate. Research in other fields has shown that individuals from ethnic minority backgrounds often report higher levels of imposter phenomena compared to their white peers. The current study did not collect data on migration background, ethnicity, or racial identity - these could be relevant variables for future research and would be worth mentioning [13, 19]. Furthermore, we cannot rule out any selection bias regarding the individuals who have responded to the questionnaire, as it is not unlikely that those have perfectionist or subservient personality types and thus are likely to have higher levels of IS, but that can neither be confirmed nor denied. Nevertheless, nearly 90% of our participants have IP, thus it is reasonable to conclude that IP is indeed very prevalent among health care professionals. This is an issue that needs to be tackled from the top, from study programs and hospitals (or other places where students do their clinical rotations) as well as by employers themselves. Empirical research on addressing imposter syndrome suggests that, although long-term studies are needed, interventions targeting IP among healthcare professionals have positive effects. Research shows how educational awareness combined with multiple strategies can build supportive environments that enhance well-being and success for health students and professionals dealing with IP [20].

Concrete strategies to address and mitigate IP include peer workshops and group discussions in the early years of training, creating opportunities for structured discussion and reflection [21]. Another strategy is to provide training and increase awareness of IP to role models or mentors who can normalize IP experiences and expectations within the healthcare profession [22]. Critically, action from leadership at the program and institutional levels are essential to create measures that identify and address IP at both individual and systemic levels. For example, including IP in institutional health and well-being plans and taking a whole-institution approach. Such leadership commitment is key to ensure the early, consistent, and ongoing interventions necessary to effectively combat IP [20, 23].

## Conclusion

The IP is highly prevalent in our study population, where almost 90% has experienced some level of IP. Furthermore, residents scored higher than consultants, yielding severity levels of significant IP as compared to moderate IP. Moreover, females are significantly more severely affected; in our study nearly one in three female residents had intense degree of IP and 70% of female consultants had significant degree of IP. We found that nurses and physicians are almost equally affected by IP, and in both professions the severity of IP decreased with increased levels of experience. There is a need to address IP both at pre-and post-graduate education of healthcare personnel. Some interventions for addressing IP and raising are offered based on the study’s findings and literature on IP.

## Data Availability

Anonymized data for this study will be available upon reasonable request to the corresponding author.
